# Hematological and Biochemical Reference Values in Healthy Captive Tigers (*Panthera tigris*)

**DOI:** 10.3390/ani11123440

**Published:** 2021-12-02

**Authors:** Daniela Proverbio, Roberta Perego, Luciana Baggiani, Giuliano Ravasio, Daniela Giambellini, Eva Spada

**Affiliations:** 1 Department of Veterinary Medicine (DIMEVET), University of Milan, Via dell’Università 6, 26900 Lodi, Italy; Luciana.baggiani@unimi.it (L.B.); Giuliano.ravasio@unimi.it (G.R.); Eva.spada@unimi.it (E.S.); 2 Veterinary Surgeon, Almenno San Salvatore, 24031 Bergamo, Italy; daniela.giambellini@gmail.com

**Keywords:** *Panthera tigris*, tigers, Bengal tigers, Amur tigers, captive, biochemical parameter, hematological parameter

## Abstract

**Simple Summary:**

The tiger (*Panthera tigris*) is the largest of the wild cats and is a pivotal member of many ecosystems and cultures. Despite a long history of concern for wild tigers, both their range and total number have collapsed: fewer than 3500 animals now live in the wild. Furthermore, an increased demand for tiger parts and consequent poaching as well as hunting of their prey has decimated populations across Asia, both in and out of reserves. All subspecies of tigers are globally endangered. Given the precarious situation of these wild felid, the health of each individual animal is of utmost importance so thorough health monitoring, including laboratory blood testing and physical evaluation, is vital. In human and veterinary medicine, hematological parameters and blood chemistry analysis are used in the diagnostic process, to establish disease processes and to monitor the progression of disease. Additionally, blood testing in wild animals provides baseline values for future studies and may help to support conservation strategies. It is extremely difficult to obtain biological samples from free-living *Panthera tigris*, therefore the values obtained from captive tigers provide very useful data. This study reports hematological and serum biochemical data in captive tigers. These results will be useful in the evaluation of physiological and pathological variations in wild and captive tiger populations. Tigers are a conservation-dependent species and require close protection because they are in high demand in the illegal trade. Development of a strong conservation ethic is urgently required for their survival. Pooling of knowledge about this species is essential if it is to be saved.

**Abstract:**

The tiger (*Panthera tigris*) is an endangered species. The health of individuals is important and any data on hematological and biochemical blood values can provide valuable information; when combined with physical assessment. This data assists in both the diagnosis of disease and some conservation strategies. The behavior of wild tigers makes it is extremely difficult to obtain biological samples from free-living subjects, therefore, data collected from captive tigers is highly valuable. The aim of this study was to provide additional information for the values of hematological and serum biochemical parameters in healthy captive tigers. Blood samples were collected from 22 clinically healthy tigers (*Panthera tigris)*. The following parameters were analyzed: glucose, urea, creatinine, alanine aminotransferase (ALT), alkaline phosphatase (ALP), total protein (TP) and red blood cells (RBCs), hemoglobin (Hb), hematocrit (Hct) and red cell indices; such as mean cell volume (MCV), mean cell Hb (MCH), mean cell Hb concentration (MCHC), platelet (PLT) and white blood cells (WBCs). The mean hematological values in our tiger population were not significantly different when compared with the same parameters in the previously studied tiger population. The mean values of RBCs and PLT were statistically significantly higher and the mean values of Hb, PCV, MCV, MCH, MCHC, and WBC were lower than the mean values obtained in previous studies on the Amur tiger. Further investigation of captive and free-living tigers is needed to identify the normal ranges for parameters in this endangered species.

## 1. Introduction

Large carnivores are among the most threatened species in the world [1]. In particular, felids are experiencing a significant contraction from their historical range [2,3]. The tiger is the largest of the wild cats [1] and is a critical member of many ecosystems and cultures. Despite a long history of concern for wild tigers, both their range and total number have collapsed: fewer than 3500 animals now live in the wild. Of these, approximately 1000 are likely breeding females [4]. As apex predators, tigers play an important role in maintaining ecological balance [3,5] but are seriously threatened by: the loss and fragmentation of about 93% of their habitat [6], reduction in genetic diversity, prey depletion and poaching [3,4,7]. The two most numerous tiger populations belong to the subspecies Bengal (*Panthera tigris tigris*) and Amur (*Panthera tigris altaica*). The Bengal tiger is the most numerous of all tiger subspecies, with a wild population of more than 2500 animals [8]. It is estimated that there are no more than 500 free living Amur tiger, which are mainly located in the Far East of Russia and in China [9,10]. 

Tigers are a conservation-dependent species and require close protection as they are in high demand in the illegal trade. The International Union for Conservation of Nature (IUCN) has classified all subspecies of tigers (*Panthera tigris*) as globally endangered [11]. Development of a strong evidence-backed knowledge base for this species is of fundamental importance to aid conservation. In the face of the decline in the wild tiger population the captive tiger population has increased in recent years, so that the number of captive tigers now exceeds that of wild tigers. Over recent decades, zoos have played a vital role in the conservation of threatened species, through captive breeding programs, research and education [12]. Alongside zoos and sanctuaries that house tigers that can join breeding and conservation programs, there also a large number of tigers that are housed in captivity for exhibition purposes only [12]. The welfare of these animals is of primary importance since they must adapt to live confined in a small and low quality habitat compared to their wild counterparts and are unable to display natural behaviors such as hunting.

Details of biological variables play a primary role in assessing the health status of both individuals and populations. Furthermore, the results of blood tests conducted in wild animals provide baseline values for future studies and may help to support conservation strategies [13]. Hematological analysis provides information on potential oxygen transportation, blood coagulation and immunity [14]. These parameters together with blood chemistry are used in both human and veterinary medicine to help diagnose diseases, to establish disease processes and to monitor the progression of disease. Given the critical situation of tigers, the health of each individual is of prime importance and laboratory blood testing, in addition to evaluation of their physical condition, is crucial in their health assessment. Due to the difficulty in detecting signs of disease in wild free-ranging or captive tigers, biological parameters are essential both for investigation and confirming diagnoses, or to assess the health and impact of disease in the individual and larger population [15,16]. To achieve these goals, there must be readily available, accurate information, on blood values from clinically healthy subjects so that abnormalities can be detected and their significance evaluated [17]. Not only must the normal range be defined, but there also needs to be an understanding of how values may vary within the normal range in response to various physiological, environmental and behavioral factors [17]. It is extremely difficult to obtain biological samples from free-living subjects, and therefore the values obtained from captive tigers provide very useful data. In addition, access to large numbers of free-living or captive individuals for testing is limited and researchers draw information on hematological and biochemical data from a limited number of individuals which makes it hard to obtain statistically significant reference values [18,19]. Therefore, each new contribution, when combined with population information, contributes to the development of a wider database.

There are few literature descriptions of hematological and serum biochemical values in wild felids. A large amount of data is available on the international database International Species Information System (ISIS) and the ZIMS database [20], which collates the results of hematological and biochemical measurements from zoos around the world [14]. Unfortunately, information about sex, age and health status of individual subjects (which may influence hematological and biochemical parameters) is not always available on this database. It is of paramount importance to produce a database containing as much information as possible. To this end, the study of a small population in which the age, gender, health status, and habitat of individuals are known, may contribute to creation of a comprehensive database that provides information about the variations in blood parameters in tigers with both physiological and pathological conditions.

There are very few studies on hematological and biochemical parameters, both in captive and free-living tigers. Hematological, biochemical and seroprotein parameters [21] have been analyzed in animals living in different forms of captivity [22] or in a wild environment [19]; while baseline hematology and biochemical values were analyzed in Amur tigers (*Panthera tigris altaica*) by Lasson et al. (2015) and Lin et al. (2020). The aim of this study was to provide an additional source of information concerning the values of hematological and serum biochemical parameters in healthy captive *Panthera tigris*, and compare these values to data from previous studies. These results will be useful for the evaluation of physiological and pathological variations in wild and captive tiger individuals and populations, and to enrich the database of parameters obtained by the analysis of other subjects in previous studies.

## 2. Materials and Methods

### 2.1. Animals and Sampling

Blood samples were collected from 22 tigers (*Panthera tigris*), including six neutered males and 16 neutered females, with ages ranging from 1 to 17 years between 2019 and 2020. All tigers were housed, in groups or individually, based on individual sociability, in various sized enclosures, rescue centers for exotic felids, zoological parks, and a circus located in northern Italy. Tigers were being immobilized for routine physical examination, minor surgical or diagnostic procedures, ocular and dental examination or vaccination administration. Tigers with any history of reported anomalies possibly associated with any pathological condition were excluded from the study. Tiger were fasted for 24 h before immobilization. All tiger received dexmetomidine hydrochloride (Dexdomitor; Vetoquinol Italia, Bertinoro (FC) Italy) at 10 µg/kg and ketamine hydrochloride (Ketavet; MSD Animal Health S.r.l., Italy) at 2 mg/kg [23]. After anesthesia induction each tiger received a clinical examination, those with any visible signs of disease, an inadequate body condition score or signs of dehydration were excluded from the group of subjects considered clinically healthy.

### 2.2. Sample Preparation

As part of the health examination, while under general anesthesia [23], blood samples were collected from the jugular, cephalic or saphenous vein of each animal. Blood was collected in ethylene-diamine tetra acetic acid (Sistema BD EDTA Vacutainer^®^, Becton Dickinson S.p.A. Milano, Italy) tubes and plain tubes (Sistema BD Vacutainer^®^, Becton Dickinson S.p.A. Milano, Italy) and serum was obtained by centrifugation for 10 min at 2500× *g*. Written owner consent for use of surplus blood samples, and use of data for scientific purposes, was obtained during consultations. The study design was approved by University of Milan Animal Welfare Bioethical Committee (Approval number OPBA 31/2019).

### 2.3. Hematological Parameters

Hematological parameters were assessed using an automated multiparameter hematology analyzer with software for animal samples (Cell-Dyn 3500 analyzer, Abbott Diagnostics Europe). The parameters measured were red blood cells (RBCs), hemoglobin (Hb), hematocrit (Hct), red cell indices; such as mean cell volume (MCV), mean cell Hb (MCH), mean cell Hb concentration (MCHC), platelet (PLT) and white blood cells (WBCs). Differential leukocyte count was made by Wright-Giemsa-stained blood smear evaluation (microscopic magnification 100 × immersion objective) with a quantitative evaluation of the percentage made in 10 fields. The presence or absence of platelet clumping was noted.

### 2.4. Biochemical Parameters

Serum samples were refrigerated at 4 °C and analyzed within 8 h of sampling. Selected biochemical analytes were evaluated on a Cobas Mira Classics Roche automated chemistry analyzer (Roche S.p.A., Mannheim, Germany) with reagents provided by Roche Diagnostics. The analytes measured were glucose (colorimetric-enzymatic method GOD-POD), urea (Kinetik ultraviolet [UV] method), creatinine (Jaffè kinetic method), alanine aminotransferase (ALT) (kinetic International Federation of Clinical Chemistry [IFCC] method), alkaline phosphatase (ALP) (kinetic DGKC method), total protein (TP) (colorimetric biuret method; Hagen Diagnostica S.r.l., Via Pratese 13 Firenze). Prior to each sample run, the analyzer was calibrated according to the manufacturers’ instructions and control samples were analyzed. Control samples consisted of various commercially available standards with values within the manufacturers’ recommended limits. Grossly hemolyzed or lipemic samples were discarded.

### 2.5. Statistical Analysis

Data were tested for normality using the Shapiro–Wilk normality test. Normal distributions, means and standard deviations were calculated, and for non-normally distributed data, medians and ranges were calculated. Due to the small sample size, the reference interval (RI) limits were directly estimated using the minimum and maximum values [24]. We compared the mean values and the standard deviation (SD), from the normally distributed tiger hematological and biochemical parameters in our analyzes, to those reported in other studies. Statistical analysis was carried out using *t*-test with *p* < 0.05, revealed statistically significant differences. Statistical analysis was performed using MedCalc Statistical Software version 15.11.3 (MedCalc Software, Acacialaan 22, Ostend 8400, Belgium).

## 3. Results

After clinical examination, 20 out of 22 captive tigers were deemed clinically healthy. For technical reasons three EDTA samples were not viable and 5 blood smears were not performed. Hematological and serum biochemical parameters were determined using the remaining EDTA samples from 17 tigers (six neutered males and eleven neutered females, with ages ranging from 1 to 17 years), along with serum samples from 20 tigers (eight neutered males and twelve neutered females, with ages ranging from 1 to 17 years) and blood smears from 12 tigers (five neutered males and seven neutered females with ages ranging from 1 to 9 years). All data, with the exception of ALT, ALP and eosinophil concentration values were normally distributed. Sex-specific and age-related differences were not analyzed because the sample size was insufficient to allow statistical evaluation. Descriptive statistics of the hematological parameters, differential leukocyte count and biochemical parameters obtained from our healthy tiger population and in populations of previous studies are given in Table 1, Table 2 and Table 3 respectively.

No significant differences were identified in the hematological values found in our tiger population compared to the Bengal tiger population previous studied [19]. Conversely, there were significant differences in the hemogram between results obtained from our population and those from previous studies regarding the Amur tiger [9,15]. The mean values of RBCs and PLT in our study were significantly higher than those obtained in Amur tiger population previous studied. In addition, the mean values of Hb, PCV, MCV, MCH, MCHC, and WBC were significantly lower than the mean values obtained in previous studies on the Amur tiger [9,15]. Mean values of neutrophil and monocyte counts in our population were significantly lower than those obtained in Amur tiger, while the mean eosinophil count was higher than that obtained in study by Liu et al. (2020). Our tiger population had the highest mean urea value amongst the compared studies. Mean creatinine concentration was lower than that found in the Amur tiger population [15], but similar to that obtained by Liu et al. (2020). Finally, in our population ALT and ALP mean concentration were higher than those found in Amur and Bengal populations previous analyzed by Larsson et al., 2015.

## 4. Discussion

The values obtained in the present study, while limited to a sample of healthy subjects of known sex, age and habitat, may help to further the understanding of healthy captive tigers and may assist in monitoring individuals and promote the conservation of this species.

The comparison of mean hematological values in our tiger population highlighted significant differences between average parameters obtained in other populations of tigers. In our study population, mean values of RBCs and PLT were significantly higher and the mean values of Hb MCV, MCH, MCHC, and WBC were significantly lower than those obtained in Amur tiger population evaluated by Liu et al. (2020). Size, number and shape of red blood cells may vary hugely among animals of different species [25], as reported in other carnivores such as dogs and wolves [15]. Although Amur tigers are members of the same cat species as our population of *Panthera tigris tigris*, the two population are not identical. The population evaluated by Liu et al. (2020), was composed only of intact Amur tigers which are the largest and tallest tigers and inhabit coniferous forests with extreme climate condition [26]. On the contrary, the population analyzed in this study is formed of a set of neutered tigers for which their subspecies are uncertain. In the dog, neutering status may affect hematological parameters, with neutered subjects having higher mean values of RBC, Hb and Hct and lower mean values of WBC, lymphocytes and neutrophils [27]. According with this report, in our study the mean values of RBC were significantly higher, and WBC significantly lower, than in the intact Amur tiger population evaluated by Liu et al. (2020). 

Therefore, the difference in number of RBC, and the values of red cell indices between our population and the Amur tiger population may be due to intrinsic differences related to the Amur subspecies or to other factors; such as variations in age, sex, diet, stress, nutritional status, altitude, exercise intensity, dehydration, splenic contraction or excitement during capture [13,15,18,22,28].

Additionally, the comparison between hematological values in captive and free-living tigers, suggests that environment has a significant effect on evaluated variables; as previously highlighted by Koubkova et al. (2002), who found that animals in hot tropical countries have higher RBC and Hb concentrations as a result of their physiological adaptation [29]. Furthermore, when comparing the data obtained in different laboratories it should be considered that several pre-analytical factors; such as different methods of analysis, sample collection and sample handling, may contribute to the variability of the results [15]. Even when using automated cell counters, the same factors may alter the results. In domestic cats the presence of rouleaux is a not uncommon finding [30]. If red cell agglutination occurs, red cell clumps may not be counted as cells, resulting in a falsely low red cell count and an elevated MCV. In this situation, MCH and MCHC may also became elevated since these indices are not directly measured but are calculated. Platelet count in our tiger population was significantly higher than reported in Amur Tiger [9]. Platelet clumping is often noted in feline blood samples [31], consequently, the irregular distribution of suspended platelets, in association with the presence of platelet aggregates, may result in inaccurate evaluation of platelet numbers which prevents conclusions being drawn. The mean PCV in our study population was significantly lower than that obtained by Larsson et al. (2015) in Amur tigers. In the absence of other hematological alterations, and of further information, it is likely this difference can be attributed to the different hydration status of the subjects.

Mean leukocyte counts were significant higher in the Amur tiger (*Panthera tigris altaica*) population than in our tiger (*Panthera tigris*) population. Changes in leukocytes may reflect both physiologic status, stress, and systemic disease, such as inflammation. The difference in mean neutrophil and monocyte values between the present study and other tiger populations, may be due to the different type of the population analyzed or to the different stress state of the animals at the time of capture and collection. Both acute stress, with response to epinephrine or norepinephrine secretion, and chronic stress, associated with increase of endogenous corticosteroids, may cause changes on the leukogram and are commonly seen in cats [32]. The Amur tiger population evaluated by Liu et al. (2021) showed a significantly higher number of neutrophil and monocyte cells combined with a lower value of eosinophil cells compared to our tiger population. This finding reflects the classic leukogram pattern as a result of higher corticosteroid concentration and may reflect chronic stress.

There were no significant differences in the mean biochemical values among tiger subspecies found across the different studies and our population, with the exception of the urea concentration, which was significantly higher in our population than in the previous studies of Amur and Bengal tigers [9,15,19], and creatinine concentration which was significantly lower in our tiger population than the concentration found by Larsson et al.(2016) in the Amur tiger population. Dehydration generally causes urea levels to rise more than creatinine levels and this is a possible explanation for the higher urea concentration values found in our tiger population. Previous studies on European wild cats and gray wolves [33,34] attributed the high urea values to the level of protein intake [9,33]. We had no information about the different diets in these populations so we can only speculate that diet may also influence the results. Serum creatinine values are correlated to the amount of muscle mass [35]. The Amur tiger is the largest living tiger subspecies, so the higher mean value found by Larsson et al. (2016) may be due to the body weight of the analyzed population. Physical activity, muscle damage and protein intake may also influence blood creatinine concentrations [18,35,36]. Additionally, chronic kidney disease is a common finding in older captive felids [37], therefore subclinical diseases may have affected the mean value of this parameter.

There are a number of limitations of this study. Firstly, although each tiger was clinically examined and screened for visible alterations and low body condition score, the history was sometimes incomplete or unavailable. This may have compromised the accuracy of categorization of animals according to health status. Furthermore, the tigers analyzed in our study could not be classified as belonging to a specific subspecies as their origin was not known with certainty, therefore the parameters we have found can only be referred to the species *Panthera tigris.* Captive wild animals included in studies often live in very different environments. These environmental factors may influence blood parameters, making comparisons difficult. Other factors, such as age, sex, neutered status, diet, habitat, subclinical conditions or the anesthetic protocol may influence the measured biochemical parameters [38]. Thus, the comparison made with the other populations studied may have been affected by different biases.

Age-related differences have been observed for the red blood cell values of Amur tigers [39] confirming findings from other studies in other tiger sub-species [40]. Sex can also influence leukogram distribution. In Amur tigers, there were significant differences in hemogram between male and female animals with increased leukocyte and neutrophil counts in males compared to females [9]. Larsson et al. (2015) reported that mean leukocyte and neutrophil concentrations were higher in males, whereas mean lymphocyte concentrations were higher in females. Due to the small number of subjects evaluated in this study, age and sex-related differences in mean values of the analyzed parameters were not assessed.

It is known whether, even within a species, the captivity status may influence the hematological and biochemical parameters [41]. Thus, the mean values identified in clinically healthy tigers may not be identical to those found in wild animals. However, since it is extremely difficult to sample from wild tigers, any contribution helps to broaden the knowledge of these basic parameters for monitoring the health status of individual animals and populations and promoting the conservation of endangered species.

## 5. Conclusions

Although carried out on a limited number of subjects, this study adds new data concerning captive tigers to the existing literature and helps to expand the database built by other authors. Hematological and biochemical values are an important tool in the assessment of the physiological status of individuals, and to monitor health status in individual wild animals and populations. These results highlight some differences between the species *Phantera tigris* and subspecies Amur and Bengal tigers. Biological, methodological, and analytical factors may have influenced the results so further investigation into captive and free-living tigers is needed to confirm the normal parameter ranges in this endangered species.

## Figures and Tables

**Table 1:** Comparative mean, median (±SD) of hematological variables in the captive tiger population of this study and of previous studies analyzing at least 10 subjects. *p* < 0.05 indicates a statistically significant difference.

Study	Our Tiger Population 2019	Liu et al., 2020	Shrivastav et al., 2012	Larsson et al., 2015
Number of animalsSub-species	17 C Undefined	112 C Amur	12 FL Bengal	13 C Amur
Parameters	Mean ± SD (min-max)	Mean ± SD (min-max)	Mean ± SD	Mean ± SD
RBC (×106/µL)	7.72 ± 0.63 (6.73–9.02)	7.13 ± 0.8 (5.35–11.05) *p* = 0.0044	7.90 ± 1.422 *p* = 0.646	7.22 ± 1.10 *p* = 0.127
Hb (g/dL)	13.4 ± 1.7 (8.5–16.7)	15.44 ± 1.8 11.6–24.1 *p* < 0.0001	12.8 ± 1.65 *p* = 3519	13.02 ± 2.63 *p* = 0.6349
PCV (%)	36.7.1 ± 3.8 (26.8–44.2)	37.10 ± 3.7 28.9–51.7 *p* = 0.6796	38 ± 2.54 *p* = 0.3117	55.49 ± 4.86 *p* < 0.0001
MCV (fL)	47.8 ± 3.9 (38.8–54.2)	52.19 ± 2.3 46.6–57.6 *p* < 0.0001	nd	nd
MCH (pg)	17.3 ± 2.04 (12.6–19.8)	21.68 ± 0.7 19.7–23.3 *p* < 0.0001	nd	18.17 ± 3.46 *p* = 0.3962
MCHC (g/dL)	36.3 ± 2.1 (31.7–40.1)	41.59 ± 1.06 39.4–46.7 *p* < 0.0001	nd	32.77 (%) ± 5.55 *p* = 0.0224
PLT (×103/µL)	259.45 ± 88 (127.5–376.5)	211.04 ± 5 63–347 *p* = 0.0024	nd	nd
WBC (×103/µL)	10.2 ± 2.7 (6.51–16.1)	11.8 ± 3.1 5.7–26.3 *p* = 0.0461	8.50 ± 1.42 *p* = 0.057	9.27 ± 2.30 *p* = 0.328

nd: not determined; C: captive; Fl: Free living; SD: standard deviation.

**Table 2:** Differential leukocyte count made by Wright-Giemsa-stained blood smear evaluation in 12 healthy captive tigers.

Study	Our Tiger Population 2019	Liu et al., 2020	Larsson et al., 2015
Number of animalsSub-species	12C Undefined	112 C Amur	13C Amur
Parameters	Mean (median) ± SD (min-max)	Mean (median) ± SD	Mean
Neutrophil (103/µL)	7.502 ± 1.4 (5.2–10.05)	10.6 ± 2.51 6.6–16.9 *p* < 0.0001	7.39 ± 2.19 *p* = 0.8689
Lymphocyte (103/µL)	2.44 ± 1.49 (1.1–6.4)	1.94 ± 1.17 0.7–8 *p* = 0.0853	1.12 ± 0.56 *p* = 0.0053
Eosinophil (103/µL)	0.35 ± 0.32 (0–0.88)	0.05 ± 0.12 0.0 ± 0.6	0.38 ± 0.23
Basophil (103/µL)	0.03 ± 0.06 (0–2)	nd	0.016 ± 0.031 *p* = 0.4258
Monocyte (103/µL)	0.16 ± 0.15 (0–0.5)	0.28 ± 0.24 0.1–1 *p* = 0.0477	0.3 ± 0.22 *p* = 0.0475

Not Normal distributed, nd: not determined; C: captive; Fl: Free living; SD: standard deviation.

**Table 3:** Comparative mean (±SD) of biochemical variables in tiger population of this study and of previous studies analyzing at least 10 subjects. *p* < 0.05 indicates statistically significant difference in mean/median.

Study	Our Tiger Population 2019	Liu et al., 2020	Shrivastav et al., 2012	Larsson et al., 2016
Number of animalsSub-species	20C Undefined	112C Amur	12 FL Bengal	10C Amur
Parameters	Mean ± SD /median Min-max	Mean ± SD Min-max	Mean ± SD	Mean ± SD
Glucose (mg/dL)	167.9 ± 58 (93–321)	176.22 ± 141.26 *p* = 0.4385	nd	188.9 ± 47.19 *p* = 0.2979
Urea (mg/dL)	75.5 ± 19.8 (37–114)	24.01 ± 8.99 15.41–77.59 *p* < 0.0001	27.90 ± 13.77 *p* < 0.0001	54.55 ± 18.35 *p* = 0.0123
Creatinine (mg/dL)	2.7 ± 0.75 (1.5–4.1)	2.74 ± 0.91 1.5–8.3 *p* = 0.8531	2.90 ± 1.03 *p* = 0.5306	3.38 ± 0.34 *p* = 0.0113
Total Protein (g/dL)	7.3 ± 0.9 (4.9–8.9)	7.48 ± 0.43 6.6–8.6 *p* = 0.1607	6.40 ± 1.88 *p* = 0.076	6.93 ± 0.46 *p* = 0.2343
ALT (U/l)	47.5 ± 24.8 (29–126)	63.9 ± 20 37–158	67.88 ± 27.84	59.27 ± 23.58 *p* = 0.2235
ALP (U/l)	31 ± 0.65 (15–122)	69.6 ± 16.3 41–115	nd	55.1 ± 35.61

Not Normal distributed; C: captive; Fl: Free living; SD: standard deviation.

## Data Availability

All study data are included in the article.
